# Blockchain Reference System Architecture Description for the ISA95 Compliant Traditional and Smart Manufacturing Systems

**DOI:** 10.3390/s20226456

**Published:** 2020-11-12

**Authors:** Erkan Yalcinkaya, Antonio Maffei, Mauro Onori

**Affiliations:** Department of Production Engineering, Royal Institute of Technology, 100 44 Stockholm, Sweden; maffei@kth.se (A.M.); onori@kth.se (M.O.)

**Keywords:** blockchain, blockchain reference architecture, smart manufacturing, ISA95, manufacturing industry, cybersecurity, IIoT

## Abstract

The next-generation technologies enabled by the industry 4.0 revolution put immense pressure on traditional ISA95 compliant manufacturing systems to evolve into smart manufacturing systems. Unfortunately, the transformation of old to new manufacturing technologies is a slow process. Therefore, the manufacturing industry is currently in a situation that the legacy and modern manufacturing systems share the same factory environment. This heterogeneous ecosystem leads to challenges in systems scalability, interoperability, information security, and data quality domains. Our former research effort concluded that blockchain technology has promising features to address these challenges. Moreover, our systematic assessment revealed that most of the ISA95 enterprise functions are suitable for applying blockchain technology. However, no blockchain reference architecture explicitly focuses on the ISA95 compliant traditional and smart manufacturing systems available in the literature. This research aims to fill the gap by first methodically specifying the design requirements and then meticulously elaborating on how the reference architecture components fulfill the design requirements.

## 1. Introduction

The ISA95 standard has a long history since 1995, when a committee of experts started working on a standard to streamline the integration of various elements of a manufacturing plant. Over time, the standard has been extended and revised repeatedly to keep pace with the new technologies, advancements, business models, and challenges in the manufacturing and production industries.

Smart manufacturing is a new breed of production concept attributed to flexibility, distributed decision, and self-feedback mechanisms, which eventually increase production efficiency and ensure fast adaptation to market conditions. These capabilities are realized with the state-of-the-art information technology (IT) advancements such as machine-to-machine (M2M) communication, industrial internet of things (IIoT), cloud, artificial intelligence (AI), and machine learning (ML).

Industry 4.0 transforms monolith ISA95 compliant traditional manufacturing systems (ISA95-CTS) to geographically distributed smart manufacturing (SMMS) units. This evolution leads to a system state where a mixture of legacy ISA95-CTS and next-generation SMMS must collaborate and work together in harmony. Moreover, the heterogeneous, interconnected, and distributed crowd of micro and monolith manufacturing facilities span across multiple geographies. Thereby, the contemporary manufacturing and production systems battle increased system complexity, which causes challenges in numerous areas.

Our previous article focused on identifying the main challenges impacting ISA95-CTS and SMMS [1]. Our research identified that systems scalability, interoperability, information security, and data quality are the primary areas where the ISA95-CTS and SMMS suffer the most. Moreover, we systematically analyzed these challenges against the blockchain technology (BCT) prospects and features. We concluded that blockchain is a key enabler technology to overcome contemporary ISA95-CTS and SMMS challenges.

In other research, we also studied on blockchain applicability of ISA95 core enterprise functions and sub-functions [2]. Our systematic blockchain assessment methodology indicated that 49 out of the 85 sub-functions and 7 out of the 12 core functions defined by the ISA95 standard are suitable for applying BCT. Some of the promising cases in this context are “Order Processing 1.0”, “Procurement 5.0”, etc.

Besides technical prospects, the reference architecture can improve supply chain viability, sustainable development in manufacturing, and social and economic inclusion in rural areas. Ivanov and Dolgui (2020) [3] researched the supply chain viability and its effects on society. Leng et al. (2020) [4] focused on the United Nations’ (UN) sustainable development goals (SDG) and highlighted how BCT could be leveraged to fulfill SDGs. Schuetz and Venkatesh (2020) [5] studied rural India and explored the social and economic inclusion through BCT based supply chain networks. Section 3.4 is dedicated to elaborate on this topic.

The researches [1,2] we have conducted so far indicate that BCT is a promising and conceptually applicable technology to empower ISA95-CTS and SMMS to overcome unique challenges. However, our literature survey revealed no reference system architecture available to realize BCT suitable ISA95 cases to support the blockchain adoption to ISA95-CTS and SMMS.

Given the above reasonings and literature gap, this research paper is positioned to define the building blocks of a blockchain system reference architecture applicable to the manufacturing industry. The level of abstraction and technical depth is carefully adjusted. Namely, the reference architecture is neither too specific nor too generic. The reference architecture promotes reusable modules to ensure applicability and consistency across the enterprise infrastructure. Moreover, the architectural components are designed with low coupling and high cohesion. Hence, high modularity is guaranteed, and the system complexity is significantly reduced. The modular system architecture is a crucial proposition to foster agility to adopt advancements in the BCT field.

## 2. Method

The spectrum for an architectural framework is broad, and the concept of software architecture has various interpretations. Many architecture frameworks, such as TOGAF and Zachman, address all phases of enterprise architectural processes, including software design, project plan, and development aspects. However, this research aims not to elaborate on all business aspects and processes of ISA95-CTS and SMMS based blockchain enterprise architecture. Instead, the focus is to define a reference architecture with system components to identify and materialize relevant blockchain technologies. The key design principle in this context is that the reference architecture is required to realize the design specification that addresses the ISA95-CTS and SMMS challenges.

The reference architecture is framed around the IEEE 1471 software architecture standard, which has three fundamental pillars [6]. Firstly, a reference architecture description must be able to identify and capture architectural concerns in the form of design specifications and requirements. Secondly, a reference architecture description must have and specify one or more systems architecture view. Thirdly, a reference architecture description must be able to realize the design specifications and requirements through the proposed architectural view(s).

The design specifications are collected from the literature and deduced from the results of our previous research articles. The reference architecture’s ultimate proposition is to overcome common ISA95-CTS and SMMS challenges and constitute a foundational design to realize ISA95 enterprise functions with BCT. The ISO25010:2011 [7] standard (Software product Quality Requirements and Evaluation), the research presented by Haoues et al. (2017) [8], and our former research article (Empowering ISA95 Compliant Traditional and Smart Manufacturing Systems with the BCT) [1] have formed the basis for the design specifications. The collected specifications are presented as functional and non-functional requirements in the following sections.

As a blueprint model, the reference architecture’s technical depth is carefully limited to neither being too generic nor too specific. Therefore, this research does not cover end-to-end business case implementations. Instead, the reference architecture defines the functionalities, system borderlines, design principles, and elaborates a high overview of architectural components (from the logical view perspective).

The BCT provides a comprehensive library of system components and infrastructure services that can be extensively customized and glued in different forms to meet a broad range of business requirements. Thereby, a BCT system architecture defined for the finance industry may considerably differ from an architecture specified for the healthcare industry. The reference architecture is specifically crafted to embody commonly available blockchain system components tailored to meet ISA95-CTS and SMMS design requirements. In other words, the reference architecture represents a snapshot of blockchain building blocks that are glued together to constitute an architectural view for the manufacturing industry.

In the technology stack, IBM Hyperledger Fabric (IHF) [9] based architectural components are reused, and an efficient blockchain consensus algorithm RAFT [10] is chosen. However, in order to reduce dependency on a single BCT provider, alternative blockchain technology providers are also discussed along with IHF in respective sections when applicable. Moreover, the reference system architecture is containerized with Docker [11]. The deployment complexity of containerization is managed by Kubernetes [12].

## 3. Reference Architecture

### 3.1. Systems Requirements

This section provides an overview of the functional and non-functional requirements, which constitutes the basis for the reference architecture proposition.

#### 3.1.1. Functional Requirements

Functional requirements in the IT domain describe system behavior. In other words, the functional requirements define the characteristics of the software and focus on what the output would be with the given input. The following points provide an overview of functional requirements deemed necessary for the reference system architecture.

**A. Use-cases:** The ISA95 standard defines 12 core enterprise functions that could be the basis for this section. In fact, our previous blockchain suitability assessment research identified 7 out of 12 core enterprise ISA95 functions suitable for BCT application [2]. However, this research aims to frame a generic reference architecture than realizing specific business use-cases. Therefore, this section only elaborates on use-cases that cover generic blockchain data communication across the ISA95-CTS and SMMS ecosystem:

1. *Use-case 1, node-to-ledger communications:* In any blockchain ecosystem, nodes continuously communicate and synchronize (read and write) data over a peer-to-peer (P2P) network to establish the distributed ledger. This typical blockchain use-case, coupled with smart-contract, can facilitate machine-to-machine (M2M) communication. As per requirement, the proposed architecture should enable node-to-ledger communications, promote autonomous industrial internet of things (IIoT), and smart manufacturing concepts through M2M capabilities.
2. *Use-case 2, ledger-to-ledger communication:* This particular use-case occurs when multiple permissioned ledgers are required to exchange data with each other. Cross subsidiaries and intercompany communication scenarios are examples of use-case 2. In order to maintain the internal ledger consistency, the proposed architecture should allow read-only data access among ledgers.
3. *Use-case 3, ledger-to-interplanetary file system (IPFS) communication:* Distributed ledgers allow blockchain actors to share data with each other. However, when the transmitted data size exceeds certain limits, sharing data through the ledger might become impractical. IPFS provides an efficient way to share oversize data for the ledger platforms. The reference architecture should be able to accommodate the IPFS communication pattern to enable the distribution of oversize data through the ledger.
4. *Use-case 4, ledger-to-external data sources (Oracles) communication:* Smart-contract and blockchain applications may sometimes need information not available on-chain. External data sources, Oracles, become handy to fetch information outside the ecosystem, and the reference architecture should support diverse data exchange patterns over Oracles.

**B. Regulatory compliance** extensively varies among business domains and indicates adherence to standards, laws, regulations, and policies. Adherence to regulatory compliance is more than a business requirement. It is, in some cases, a legal obligation. Therefore, the reference architecture should provide technical mechanisms and capabilities to ensure regulatory compliance across the blockchain ecosystem.

**C. Data integrity and signing** are cybersecurity functions that ensure that the data is consistent, accurate, and not tempered during the entire lifecycle. Besides, signing also provides source authentication and non-repudiation of origin. Thereby, the reference architecture should support a broad range of data integrity and digital signature schemas to address on-chain and off-chain data integrity and signing challenges.

**D. Automation** is a critical capability to increase efficiency, reduce human errors, and enable new technology adaptation in any business area. The ISA95-CTS and SMMS are no exception to this, and the reference architecture should be able to promote and allow manufacturing automation capabilities without any compromise.

#### 3.1.2. Non-Functional Requirements

Non-functional requirements encompass a wide range of operational behavior of a system and focus on how a software fulfills its functions. Non-functional requirements are not directly related to the specific business use-cases. However, they are foundational system capabilities to make sure that the system operates as designed. Although the spectrum of non-functional requirements can be extensive, we have identified the following as the most crucial system capabilities that the reference architecture is required to embody. In fact, our previous research article identified the majority of the non-functional requirements listed below as the significant pain points impacting the ISA95-CTS and SMMS [1].

**A. Scalability** incorporated with elasticity is a property ensuring that a highly loaded system can seamlessly grow up to handle the increasing load and shrink down to return to the normal state. The ISA95-CTS and SMMS ecosystem constitute a broad scope of devices and systems with varying computational capabilities where scalability becomes a crucial design requirement [13]. Therefore, the critical components of the reference architecture should horizontally and vertically scale to be able to meet the performance requirements stated in the following section.

**B. Performance** can be measured and evaluated in several ways. Bamakan et al. (2020) [14] performed a literature survey and compiled an extensive table with different performance evaluation criteria for blockchain performance benchmarking. However, we pick the two most featured system performance criteria for our reference architecture design work: System throughput and latency.

Each ISA95 automation pyramid level [15] has a varying response time. For instance, the automation level 0 actors (sensors, field devices, and IIoT) operate in near-real-time. However, on upper layers where human intervention is involved, the operational time frame may extend up to weeks and, in some cases, even months. Thereby, specifying the optimum level of latency requirement is not straight forward.

As specified in “Use case 1”, enabling process automation through M2M capabilities with constant feedback mechanisms is crucial to ensure manufacturing agility. Rondon et al. (2017) [16] presented latency requirements for closed and open-loop industrial process automation applications. They highlighted that the required update time for IIoT is 100 to 5000 milliseconds (ms). Likewise, according to Ferrari et al. (2020) [17], ordinary IIoT devices connected to MQTT need 50 to 300 ms on average for a round trip (including processing time). Thereby, the reference architecture should ensure latency below 300 ms, fulfilling both automation and processing latency requirements.

The system throughput requirements extensively vary on the business needs and the size of the enterprise ecosystem. However, a wide-scale blockchain system serving a mid to large-scale manufacturing corporation should manage 6000 to 8000 transactions per second (tps). In fact, Sund et al. (2020) [18] analyzed a global furniture manufacturing company, IKEA, and specified the throughput requirements as 7840 transactions per second.

**C. Interoperability** has many folds in modern enterprise architecture stack, including the blockchain-based applications. According to Abebe et al. (2019) [19], blockchain applications are built upon a four-layered abstraction model. These abstraction levels are in technical, syntactic, semantic, and governance domains. The proposed reference architecture should be able to ensure seamless interoperability among systems integrating and communicating with the ledger on all four domains mentioned above. Thereby, not only the legacy devices but also next-generation technologies such as cloud, ML, AI can seamlessly integrate and understand each other.

**D. Data quality** is measured with 15 distinct data characteristics specified by ISO25012 standard [20]. The ISA95 specification consists of 12 enterprise core functions and 85 sub-functions that demand and fulfill data quality characteristics with varying controls. Therefore, instead of framing a standard quality requirement (that would not apply for all cases anyways), the reference architecture should define a set of quality violation policies as unified system behaviors for different quality control situations.

**E. Information Security requirements:** The importance of information security has become significant with the recent cybersecurity events. Furthermore, international institutions and regulators extend the existing standards and policies to embody new security requirements. Thus, the reference architecture should incorporate the following built-in native security features to meet the regulatory requirements and minimize cybersecurity risks.

- **Data protection** is a fundamental security design requirement that is expected to be fulfilled by any modern IT system regardless of the business domain. The data protection becomes even more crucial for the blockchain-based systems, which copy the ledger content among several nodes. The distributed architecture with vast data replication imposes robust data protection mechanisms. Thus, the reference architecture should be equipped with technical controls to protect the data at rest, in transit, and during processing from unauthorized access.
- **Logging, traceability, and auditability** are essential security capabilities that ensure transparency and accountability. Auditability and traceability allow legitimate parties (3rd party verifications through auditors) to track the history of a user, system, and transactional events. Extensive logging mechanisms functioning on various system levels ensure auditability and traceability. Hence, the blockchain-based reference should be able to provide traceability and auditability along with robust, secure, and scalable logging services.
- **Availability** ensures that the system is accessible when desired under any circumstances. Highly available systems are fault-tolerant and robust to maintain the operational state. In the manufacturing industry, high availability plays a significant role. Although the criticality levels and maximum tolerable downtime might differ, most operational functions can be classified as mission-critical. Consequently, the reference architecture is expected to fulfill high availability requirements.
- **Identity and access management** mechanisms provide technologies to identify a broad range of ISA95 actors (i.e., subjects in IAM terms) and ensure that only privileged actors can access enterprise resources. Modern manufacturing organizations rely upon complex business processes that are interconnected with a variety of internal sub-organizational units and external partner associations. Therefore, the reference architecture should provide capabilities to identify, authenticate, and authorize a broad spectrum of unique actors spread across all ISA95 automation layers and dispersed on numerous organizational structures. Moreover, the reference architecture should have technical mechanisms to ensure fine-grained authorizations.

### 3.2. Reference Architecture Logical View and Components

**Figure 1:**
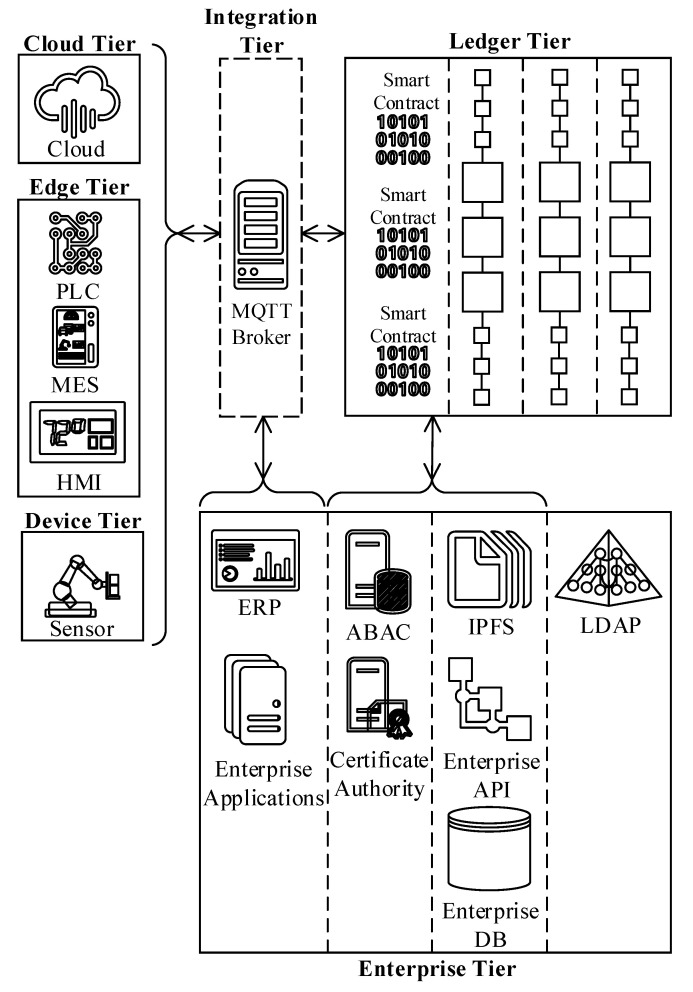
Reference architecture logical view and components.

The reference architecture proposition manages the ISA95-CTS and SMMS ecosystem complexity with a layered approach. The logical view is composed of six main tiers, and Figure 1 demonstrates the tiers with corresponding actors and subsystems along with their interactions among each other on a conceptual level. Modern system solutions are designed with high scalability and elasticity. Thereby, considering the versatility of the ecosystem, containerized run-time environments (such as Docker [11]) strengthened with automated deployment and orchestration tools (such as Kubernetes [12]) can be utilized to manage the complexity. The containerized approach, where each tier is administered and separated by a corresponding namespace, enables hybrid (on-prem and cloud) deployments.

The following bullets briefly explain the tiers and high-level architectural components illustrated in Figure 1. The realization of the non-functional and functional requirement section covers each architectural component in detail (i.e., technical specifications, interdependencies, etc.).

- *Device tier* corresponds to the lowest ISA95 automation layer (level 0) where the field devices (i.e., cyber-physical systems including sensors, robots, actuators, etc.) are placed. The device tier traditionally has minimal processing power and may contain a wide range of next-generation (e.g., IIoT) and legacy (e.g., Modbus) devices. Thus, the device tier elements would need software agents and an integration layer to communicate with the ledger successfully.
- *Edge tier* encapsulates the ISA95 level 1, 2, and 3 devices (i.e., programmable logic controller (PLC), manufacturing execution system (MES), human-machine interface (HMI), supervisory control and data acquisition (SCADA), etc.) that process the data feeds from the underlying layers and provide various process automation capabilities. Like the device tier, this tier also contains a broad spectrum of hardware appliances with varying abilities. Thereby, next-generation devices supporting TCP/IP based communication protocols can directly talk to the ledger, whereas legacy devices may require a software agent and an integration layer to exchange data with the blockchain.
- *Cloud tier* contains the cloud resources such as AI, ML, and IIoT hub. Some cloud services might require intermediaries to talk to the ledger, whereas others might directly talk to the blockchain. Thus, the integration layer is optional for the cloud tier.
- *Integration tier* plays a vital role in ensuring interoperability among various ISA95-CTS and SMMS layers. Because some systems, such as IIoT devices, are customizable by design and capable of exchanging data directly with the ledger tier, the integration tier is optional. Thus, the demarcation of this tier is marked with dashes. Although there are a few alternatives, the reference architecture adopts Message Queuing Telemetry Transport (MQTT) protocol for the integration tier. The rationale behind this design decision is that MQTT provides versatile and flexible data conversion capabilities with built-in data adaptors to support out of the box integration capabilities. Moreover, the MQTT protocol embodies an extensive library of numerous communication protocols and messaging standards that are useful to automate system integration.
- *Enterprise tier* is divided into four sub-regions as follows:
  (a) The first sub-region (Figure 1) consists of applications that belong to the ISA95 automation pyramid level 4 actors such as ERP and other enterprise applications.
  (b) The second sub-region embodies off-chain infrastructure services that are directly consumable by the blockchain IAM. These infrastructure services comprise enterprise certificate authority (CA) and attribute-based access control (ABAC) system components.
  (c) The third sub-region consists of off-chain data sources that are consumable through data oracles. These data sources can be in many forms, including, but not limited to, IPFS, enterprise APIs, and enterprise DB instances.
  (d) The fourth sub-region accommodates an enterprise directory service, LDAP, which is not directly consumable by the ledger. LDAP supplies additional identity attributes to enrich the basic identity profile produced by the CA.
- *Ledger tier* represents the reference architecture’s backbone, and obviously, the rest of the tiers are designed to support the ledger tier. The target group for the blockchain reference architecture is the manufacturing industry, for which the information security, privacy, and system performance requirements are at the highest level. In order to meet these requirements, the reference architecture is based on a permissioned blockchain solution where access to the blockchain ecosystem is restricted.

Modern ISA95 manufacturing enterprises rely on sophisticated organizational arrangements, and business engagements span around the globe. Thereby, restricting access to enterprise-level may not be sufficient to fulfill the business, security, and regulatory requirements. In other words, the reference architecture should be able to support fine-grained access control mechanisms to ensure privacy and confidentiality even within the same organization. The reference architecture meets this requirement by establishing dedicated ledger instances (channels) with specific access control lists (ACL). On some rare occasions, when even finer-grained access control is required, the reference architecture consults an off-chain policy decision point (PDP) relying upon attribute-based access control (ABAC) mechanism.

The actors belonging to the device and edge tiers have minimal processing and storage capacity. Similarly, most enterprise tier applications are proprietary systems that are not customizable to accommodate a chain structure. Therefore, the heterogeneous nature of ISA95-CTS and SMMS ecosystem leads to an isolated system design where the blockchain content is not replicated outside the ledger tier. In other words, the ledger tier does not distribute the ledger content nor share the blockchain transaction verification operations with the remaining tiers. The ledger isolation might be perceived to conflict with the blockchain distributed system design philosophy. However, this restriction only concerns tier abstraction. The containerized design approach allows the reference architecture to allocate blockchain node instances wherever needed. In fact, in order to ensure high availability and low latency, each tier should contain multiple full-nodes within the same network zones where the respective ISA95 actors are segmented.

### 3.3. Realization of Systems Requirements

#### 3.3.1. Realization of Functional Requirements

The following subsections cover the realization of functional requirements.

**A. Use-cases:** The ISA95 standard defines 12 core enterprise functions that could be the basis for this section. In fact, our previous blockchain suitability assessment research identified 7 out of 12 core enterprise ISA95 functions suitable for BCT application [2]. However, this research aims to frame a generic reference architecture than realizing specific business use-cases. Therefore, this section only elaborates on use-cases that cover generic blockchain data communication across the ISA95-CTS and SMMS ecosystem:

As specified by the requirements sections, this research work does not focus on specific ISA95-CTS and SMMS business use-cases; instead, it only elaborates on the following generic blockchain communication use-cases.

**Figure 2:**
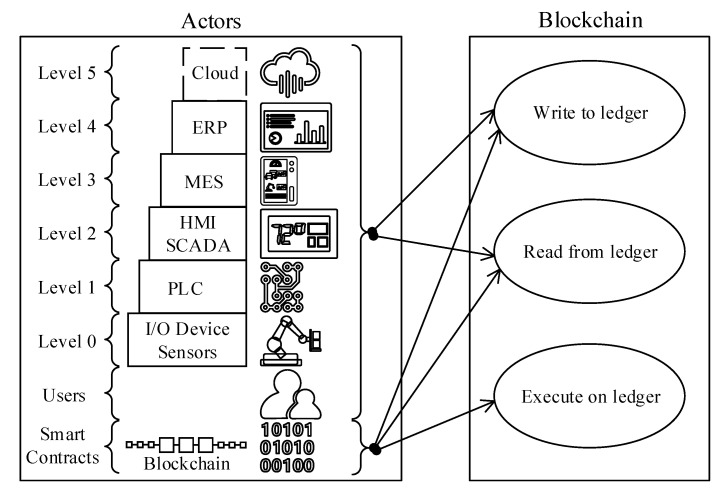
Use-case 1, node-to-ledger communications.

**Figure 3:**
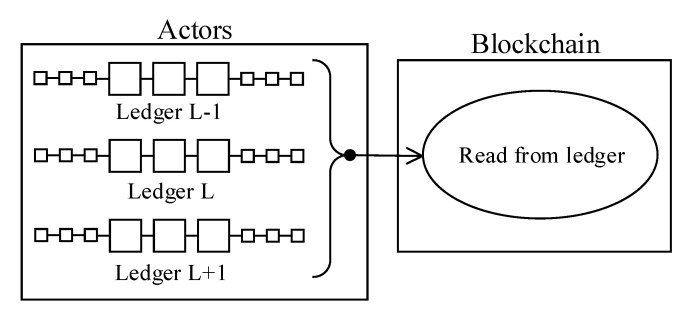
Use-case 2, ledger-to-ledger communications.

**Figure 4:**
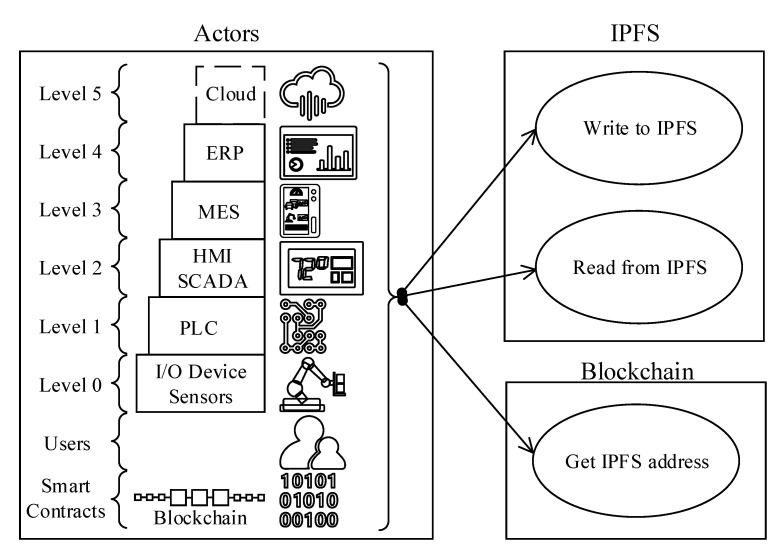
Use-case 3, ledger-to-interplanetary file system (IPFS) communication.

**Figure 5:**
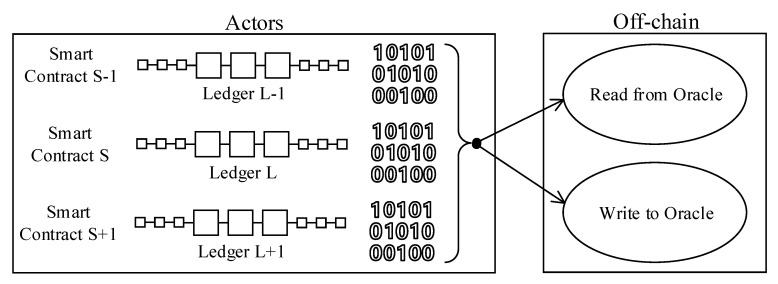
Use-case 4, ledger-to-external data sources (Oracles) communication.

1. *Use-case 1, node-to-ledger communications:* As per the design requirement, the proposed blockchain reference architecture should enable M2M communications and autonomous decision mechanisms. The ISA95-CTS is built upon a classical server-client application architecture, and the actors described in the automation pyramid (Figure 2) can generally only talk to one layer above or below. Given the ISA95 standard’s hierarchical structure, achieving M2M communications within the ISA95-CTS boundaries is not a straightforward integration pattern. Nevertheless, a ledger-based data communication pattern can overcome the hierarchy problem. This is because the ledger acts as a data integration layer that enables any ISA95 actor to directly communicate with any other actor within (or even beyond) the ISA95 ecosystem. For instance, a level 4 enterprise resource planning (ERP) application can directly talk to a level 0 sensor. Likewise, an IIoT device, such as a sensor, can communicate with another IIoT device on the same automation level without requiring to cascade the data through upper layers. Also, a smart-contract can initiate autonomous M2M actions by coupling sensors and actuators located on level 0. In fact, Afanasev et al. (2018) [21] studied M2M communication in the SMMS context and explored the smart-contract features with a proof of concept (POC) implementation. Their research article also focused on the M2M micropayments concept through blockchain.
  Figure 2 demonstrates “Use-case 1,” enabling all ISA95 actors to directly read and write to the blockchain. Because all designated actors can talk to each other through the blockchain, device interoperability becomes a key enabler to realize the proposed architecture. The following sections address interoperability requirements.

2. *Use-case 2, ledger-to-ledger communications:* Cross-chain interoperable data exchange and cross-chain inter-executable smart-contract concepts are open research areas in the literature. There are a limited number of articles available in the literature focusing purely on this domain [22,23]. Ledger-to-ledger communication use-case typically occurs when two or more entities happen to utilize separate blockchain installations that are expected to operate or execute a cross-chain smart-contract. Although there are many challenges to realize the ledger-to-ledger use-case, the most prominent issue is maintaining internal data state consistency when the data is modified or created across multiple ledgers. Given the above restrictions, the reference architecture only partially fulfils the ledger-to-ledger communication use-case (Figure 3). Namely, ledgers can read data from each other but not allowed write, create or update data residing on another ledger instance.
  Data oracles enable smart-contracts to read off-chain data, including other blockchain instances. Therefore, a smart-contract allocated on a source ledger can access the target blockchain through a specially crafted oracle (Figure 3).

3. *Use-case 3, ledger-to-interplanetary file system (IPFS) communications:* The block and ledger sizes are the most crucial system properties that directly impact the blockchain scalability and performance. Qui et al. (2019) [24] studied various blockchain platforms relying on IPFS and reported significant performance improvements when IPFS is adopted for large data sets. The reference architecture is designed to reduce the blockchain footprint by hosting oversize data through IPFS based file share attached to the blockchain P2P network.
  “Use-case 1” above explained how the ISA95 automation pyramid is flattened when BCT is adopted. A similar design pattern also applies to the IPFS data communication scenario. Therefore, various actors shown in Figure 4 can directly read and write to IPFS via P2P network protocols (given that relevant access rights are granted).
  The “Use-case 3” can be realized with an operational data policy that is implemented as a blockchain smart-contract. The suggested policy can define thresholds, and if a file or transaction content is above the limit, then the blockchain operating system makes the actor write the file content to IPFS. Next, the file hash (acting as the pointer to the original file) is generated and inserted as a new block content to the blockchain.
  When an actor needs to access the file, first gets the file address on IPFS from the blockchain, and then the actor accesses the address location to read the file content.

4. *Use-case 4, ledger-to-external data sources (Oracles) communications:* ISA95-CTS and SMMS ecosystem are composed of several systems, including legacy and externally hosted applications. Not all pieces of this complex environment are compliant with the blockchain architecture. Thus, off-chain data sources will always contain crucial information to on-chain applications and blockchain smart-contracts. The data outside the blockchain system are made available to the blockchain environment through data Oracles. Rest interfaces, SQL database connectors, file parsers are just a few data sources that blockchain oracles can read and fetch data. Likewise, off-chain applications might also need to get data from the distributed ledger. Reverse oracles become handy to emit (write) the blockchain state data to the external world [25]. Figure 5 visualizes “Use-case 4” as follows.

**B. Regulatory compliance:** Modern SMMS extend the manufacturing capabilities from a single monolith enterprise towards geographically distributed manufacturing units. This complex organizational structure leads to challenges in fulfilling regulatory compliance across different country jurisdictions. Moreover, regardless of geographic dispersion, ISA95-CTS is subject to a broad spectrum of manufacturing standards such as “ISO 9001” and “IEC 61646”.

**Figure 6:**
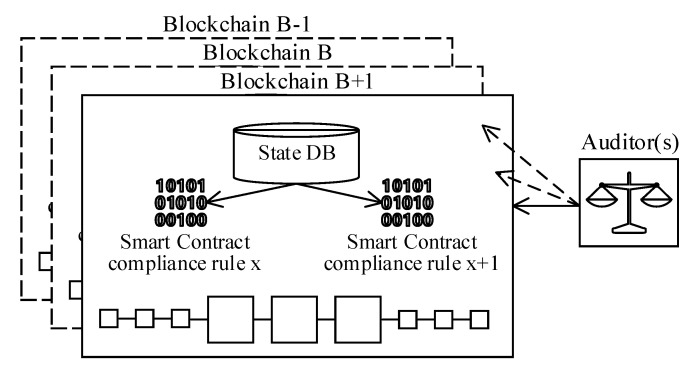
Distributed blockchain-based compliance architecture components.

Smart-contracts convert contractual terms and conditions to a piece of code that autonomously executes in a tamper-proof run-time environment on a blockchain. Hence, independent and unbiased decision mechanisms are guaranteed. Zhang et al. (2019) [26] studied these unique characteristics and proposed a blockchain-based distributed compliance architecture that is composed of the following system components (Figure 6):

1. Smart-contract code (implementation of compliance requirement)
2. State DB
3. Auditor interface

As discussed in the following sections, the reference architecture is designed across multiple isolated channels (dedicated blockchain instances). Because each channel realizes a different business use-case, the compliance adherence requirements may vary across channels. Likewise, the number of smart-contract implementations might differ from channel to channel. Moreover, a dedicated database is allocated to each channel to isolate and store compliance state parameters. Lastly, regulators and auditors are provided with a read-only interface to perform compliance verification activities.

**C. Data integrity and signing:** The manufacturing reliability, supply chain consistency, document originality, and transaction legitimacy can be ensured with properly implemented integrity and digital signing capabilities. Given the topic’s versatility, the reference architecture realizes data integrity and the signing in two dimensions.

In the first dimension, on-chain data integrity and signing operations are elaborated. The blockchain is comprised of tamper-proof data blocks that are linked to each other with a hash-based pointer. The hash is uniquely calculated from the block content with the help of one-way mathematical functions, and even if one bit is altered from the data block; a completely new hash value is produced. Hence, the immutability (i.e., integrity) of on-chain data is ensured on the highest possible level.

The digital signatures provide data integrity, source authentication, and non-repudiation of origin. ISA95-CTS and SMMS ecosystem is composed of a wide range of systems. Fang et al. (2020) [27] surveyed all prominent digital signature algorithms in the blockchain context. The reference architecture offers pluggable cryptographic library extensions to support various blockchain signature algorithms studied by Fang et al. (2020) [27]. The scope of this paper is not bound to a specific business scenario or algorithm. In order to demonstrate the flexibility of the reference architecture to support different business needs, the following three sample use cases are exemplified.

1. The edge and device tiers contain cyber-physical and IIoT systems that have limited processing power. Thereby, the transactional data can be signed with less process-intensive cryptographic signature algorithms to lower the system overhead. Aggregate signatures are considered the best fit for low processing ecosystems because the algorithm can compress n signatures of n messages belonging to n persons into one compact signature.
2. Some business flows, such as financial use cases, require a higher level of assurance with separation of duties. In this case, multi-signature schemas can provide the desired level of trust and security.
3. Preserving privacy without compromising authentication and non-repudiation of the signer might be necessary for some business scenarios. In the ISA95 “Product Shipping Admin 9.0” business function, for instance, the receiving customer can verify and track the shipping status without revealing the actual identity of the person who has shipped the goods from the factory. Sanchez et al. (2018) [28] focused on a similar problem in the context of the internet of things (IoT). They proposed a privacy-preserving data signature and verification algorithms based on the NI-ZKP concept. Sanchez et al. demonstrated and mathematically proved that the NI-ZKP concept ensures authentication and non-repudiation without revealing the underlying identities.

In the second dimension, off-chain data integrity and signing operations are elaborated. Gaetani et al. (2017) [29] identified and categorized major threat actors against the corporates’ data integrity. These actors can be in two orders: Externals such as hackers, and internals, such as employees, contractors, and federated business partners. Hashing and signing are powerful cryptographic operations to ensure data integrity with the condition that the hash and signature values are kept in a tamper-proof environment. Traditional blockchain applications store the data on the blockchain along with the hash and signature values, which ensures immutability and integrity through the linked data structure. The drawback of this approach is that the ledger grows too fast. Especially considering a large corporation with many workstations and servers with billions of files in various sizes, replicating all off-chain data to a ledger is not scalable.

The reference architecture addresses the integrity challenges for the off-chain data in a unique way. Instead of fully onboarding and replicating individual data elements (files, documents, etc.) to the ledger, the reference architecture proposes only to protect the data hash and signature values. This concept can be exemplified as follows. According to the ISA95 functional model, the “Quality Assurance 6.0” function feeds the production standards to the “Production Control 3.0” function. The production standards constitute detailed information on all aspects of production processes. Hence, the file containing the standards might allocate significant space on disk and cannot be feasible to distribute over the ledger. The reference architecture realizes this scenario by distributing big files over alternative ways (e.g., P2P, network share, etc.) and protecting the integrity digests (hashes) to the files on the blockchain (similar to “use case 3”). Once the data file is received, the “Production Control 3.0” function generates the file hash and compares it with the hash value protected on the ledger. In case more security is demanded with source authentication and non-repudiation, file signature values signature can be secured on the blockchain. Besides the foundational security features, sharing the signature values via the ledger also mitigates man-in-the-middle attack risks.

**D. Automation:** Because automation and autonomous decision mechanisms are qualified as the most distinctive characteristic of SMMS, the reference architecture is required to embody strong automation capabilities, which can be realized with the help of the smart-contracts as follows. First and foremost, the smart-contracts execute the pre-implemented application logic in a tamper-proof environment. With this given property, deterministic process automation is ensured. Second, not all business flows and processes are expected to operate inside the blockchain ecosystems. In fact, corporates adopting the ISA95 standard are large enterprises composed of a broad range of systems. In such cases, the smart-contracts can emit event messages via reverse oracles, contributing to the autonomous decision mechanisms outside the blockchain ecosystem.

#### 3.3.2. Realization of Non-Functional Requirements

The following subsections cover the realization of non-functional requirements.

**A. Scalability:** The scalability requirements in IT apply to two resource types, processing power and storage capacity, and can be fulfilled in two dimensions. The vertical scalability concerns expanding the existing resources (scaling-out), whereas the horizontal scalability relates to adding more individual elements (scaling-up) to share the workload.

The blockchains are loosely coupled distributed systems capable of rapidly scaling-up on the horizontal axis to increase the processing power. The reference architecture is no exception to this. Furthermore, the reference architecture is designed on a fully virtualized container environment that can provide a high degree of horizontal and vertical scalability. The containerized architecture is studied in the literature too. Ioini and Pahl (2018) [30] suggested a container-based trustworthy blockchain system to manage the IoT-centric edge computing infrastructure solutions. Ioini and Pahl also implemented a POC application based on IHF blockchain and Docker container technologies.

Containerization is not exclusive to the IHF and can easily be tailored to a whole range of blockchain platforms. In fact, Macdonald et al. (2017) [31] surveyed several BCTs and highlighted that Eris (a hybrid form of Tendermint and Ethereum) blockchain platform leverages Docker-based containerized system components.

The scalability of storage capacity is correlated with the size of the ledger. The blockchains are immutable and append-only databases. Therefore, the size of the ledger always grows over time. Hence, blockchain system architects design the block structure to reduce the ledger footprint as small as possible. The reference architecture addresses the size constraint by storing big data, such as media files, on a separate network storage unit, IPFS. However, even with delicate system design, nodes and peers will eventually run out of space. In such scenarios, containerization would allow the reference architecture to scale out the storage capacity quickly.

Elasticity is characterized by the reaction speed to meet the growing and shrinking system scalability requirements. The elasticity of a system can be automated with autoscaling capabilities. Kubernetes, the container orchestration tool chosen for the reference architecture, is able to scale workloads horizontally and vertically. Depending upon the configuration, when the predefined CPU utilization target is reached, the Kubernetes autoscaler tool can add new pods to the group of workloads to maintain a consistent performance level. Likewise, the storage capacity can be expanded vertically with the Kubernetes PersistentVolumeClaim request, allowing dynamic storage management.

**B. Performance:** The performance of the reference architecture is measured with system throughput and latency. The performance of a blockchain-based system depends on the deployment topology, consensus algorithms, and block size.

Publicly available blockchain technologies are not the best alternative when high-performance is demanded. For instance, the Bitcoin infrastructure’s average system throughput is around a few transactions per second (tps). Meanwhile, the latency can extensively vary from 10 min up to even a few hours in some extreme cases. Permissionless blockchain technologies sacrifice on performance front in order to be able to validate the correctness of anonymous transactions with computationally complex consensus algorithms, such as proof of work (PoW).

On the other hand, permissioned alternatives rely on trivial consensus protocols such as RAFT or PoS. Du et al. (2017) [10] compared five popular blockchain consensus algorithms and demonstrated that permissioned ledgers with RAFT consensus algorithm could easily exceed 10,000 tps throughput. Sukhwani et al. (2018) [32] benchmarked various blockchain operations for the IHF permissioned ledger architecture and observed the system latency between 2.56 ms to 208.30 ms. The same research also assessed the block size (number of pending transactions per block). While increasing the block size positively affects throughput (when there are four or more ordering nodes), the latency is negatively affected. Thus, there is a subtle balance among block size, latency and throughput. Sukhwani et al. also demonstrated that adding more processing power (CPU quantity) contributes to lower the latency.

Several alternative blockchain platforms offer equivalent and, in some cases, even better throughput and latency measurements. For instance, according to a comparative distributed ledger analysis [33], EOS and Corda blockchain platforms’ latency is equivalent to the IHF proposition. Likewise, hashgraph based distributed ledger platforms (such as Hedra) offer exceptionally high throughput and exceed the 100,000 tps barrier [34].

The reference architecture design specification requires 300 ms maximum latency with a throughput of 6000–8000 tps. These requirements can be met with a permissioned BCT employing a trivial consensus protocol RAFT. The reference architecture should incorporate at least four ordering services with eight CPU cores each, and individual block size should be limited to a maximum of 80 transactions.

**Figure 7:**
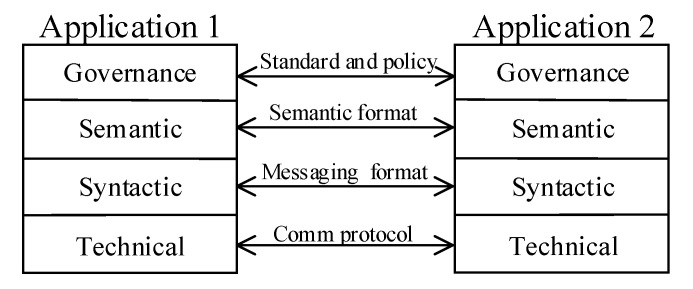
Blockchain application interaction model.

**C. Interoperability:** Abebe et al. (2019) [19] explained the enterprise blockchain application interactions and interoperability with a four-layered abstraction model (Figure 7). The reference architecture blockchain ecosystem comprises a broad range of actors, all of which are required to be compliant with the four-layered abstraction model. The following lines elaborate on how the reference architecture fulfills the systems interoperability design requirement on each blockchain application interaction layer.

The Message Queuing Telemetry Transport (MQTT) is a TCP/IP network protocol that is formalized with the ISO/IEC 20,922 standard. MQTT establishes a unified integration platform for a wide range of communication protocols to talk to each other. MQTT is based on a publish/subscribe integration pattern to exchange messages through a message broker that comes out of the box with several data adaptor types to convert various wire protocols and message formats. Given these capabilities, MQTT is a perfect match to ensure interoperability on technical and syntactic interaction layers among all the ISA95 actors and the blockchain. Ramachandran et al. (2018) [35] researched a novel MQTT-based distributed integration platform to guarantee various IoT devices’ interoperability to talk to each other over a ledger instance.

The ISA95 standard [15] defines (booklet 2, 4, and 5) interfaces, data object models, and standard business transaction structures. Therefore, systems compliant with the ISA95 standard are semantically interoperable with each other. However, an intermediary layer for semantic conversation would be needed for the incompliant actors with the ISA95 semantic standard. MQTT platform would become handy in developing specially crafted ISA95 semantic format transformation adaptors to ensure the ISA95 compliancy.

Data governance is all about standards and formal policies. Therefore, as elaborated on the “Regulator compliance” section, the interoperability on the governance layer can be ensured and realized with the smart contracts.

**Figure 8:**
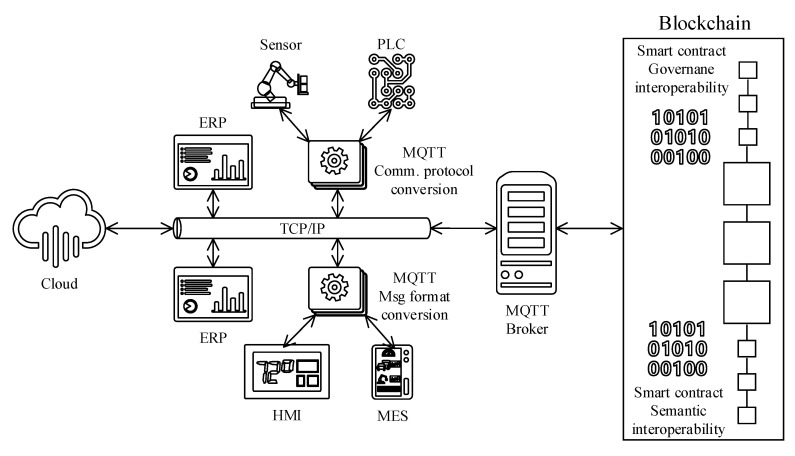
Interoperability components for the reference architecture.

Figure 8 illustrates various ISA95 actors and architectural components that realize the interoperability for the reference architecture.

**D.Data quality:** The reference architecture defines quality violation policies as unified system behaviors across the distributed ledger ecosystem. These behaviors can be realized in three ways, as follows [36]:

- As part of application logic
- Extension of consensus protocol
- Policy-based violation behaviors enforced via smart-contracts

We assessed the third option, policy-based violation behaviors enforced via smart-contracts, as the most viable option for the reference architecture. The rationale behind this design decision is that smart-contracts ensure autonomous and unbiased policy enforcement that cannot be circumvented. Moreover, adopting new business requirements and extending the existing smart-contract are relatively easy to operate. For instance, once a revised action is available, all actors depending upon the particular smart-contract can seamlessly start using the latest version. On the contrary, if the violation reactions are incorporated into each application, even a small revision will be a significant maintenance task. Similar restrictions on scalability and flexibility apply if the violations are combined with the consensus protocols [36].

In order to streamline application responses, the reference architecture adopts the following data quality violation behavioral policies that are an extension of similar policies suggested by Cappiello et al. (2019) [36].

- *Accept and log on violation policy* is useful in occasional cases when the quality parameter is not within the expected range. For instance, some sensors might produce unreliable data that does not conform with the predefined quality characteristics during calibration and configuration phases. The application that is aware of the unusual situation can choose to accept the violation and register an event to the system log files.
- *Alarm on violation policy* concerns the default behavior when the quality parameter is outside the boundaries. In this case, the parameter value is rejected, and a warning is generated to inform the operator.
- *Raise an event on violation policy* incorporates actions to handle a violation. In other words, when a quality violation occurs, the system takes predefined measures to correct the situation. For instance, if a malfunctioning robotic arm causes a production line to produce distorted goods, the violation policy can immediately halt the production line to prevent further damage.
- *Defer decision on violation policy* is useful when a single violation is not sufficient to make a concrete decision. In other words, the system does not respond to a particular quality issue until a certain number of repetitions of the same violation occurs.

The reference architecture can centrally operate and manage the listed violation policies as smart-contracts. The applications relying upon data from the ledger can choose to execute an appropriate smart-contract instance on a use-case basis.

**E.Information Security:** The following sections elaborate on how the information security requirements are realized for the blockchain-based ISA95-CTS and SMMS reference architecture.

**a.**  **Data protection**

The data on a blockchain can be in three states. Namely, data in transit, data while in use, and data at rest. The reference architecture is designed to protect the data in all three phases, as follows [1].

***Protecting data in transit*** is relatively more straightforward than the other two states. Almost all modern IT systems, including SMMS, natively support TCP/IP protocol. Thereby, the transit data can be protected with encryption via transport layer security (TLS) protocol. However, traditional ISA95 compliant manufacturing organizations are composed of a broad spectrum of cyber-physical devices, some of which rely on outdated or tailor-made communication protocols that cannot be protected with TLS out of the box. The reference architecture is designed to transform proprietary protocols over the integration tier with MQTT.

The following bullet points briefly elaborate on how the data in transit is secured for various ISA95 actors with different capabilities.

1. ISA95 actors supporting TCP/IP and HTTPS protocols can connect to the MQTT integration bus over TLS protected communication channel.
2. ISA95 actors supporting TCP/IP but not HTTPS protocols can connect to the MQTT service bus through a reverse proxy that hides the insecure endpoint address. The ISA95 actor is configured only to accept requests from the reverse proxy. Thereby, interacting directly with the actor through an insecure channel is not possible. The reverse proxy supports TLS; thus, the communication line towards the MQTT integration bus is fully encrypted.
3. ISA95 actors not supporting TCP/IP protocols are hardwired to an MQTT publisher with protocol conversion capability. This integration pattern enables non-TCP/IP compliant actors to be able to communicate with the rest of the actors with an encrypted TLS tunnel.
4. Data communication among suppliers, subsidiaries, and geographically dispersed manufacturing facilities are protected with secure tunneling protocols such as IPSec VPN.

***Protecting data while in use*** is usually a disregarded topic in IT. In fact, even modern systems store the data in memory in an unencrypted format. Moreover, hardware and software solutions are designed to process unencrypted data. These drawbacks have recently been mitigated with a newly developed technology that relies on a containerized, trusted execution environment (TEE). It is possible to enable TEE with either hardware-based (e.g., Intel software guard extension—SGX) or software-based (e.g., Microsoft Hyper-V virtualization-based security—VBS) providers. Both alternatives segment the data run time from the rest of the operating system with isolated containers (i.e., enclaves). Furthermore, all data outside the enclave is fully encrypted, and a third party formally verifies the code segment that processes the data inside the enclave. Thereby, the TEE technology mitigates recently discovered run-time attacks such as memory scraping and malicious process snooping while data is in use [37].

Since hardware-based enclaves offer better isolation and higher performance, the reference architecture is designed to harness hardware-based TEE to process sensitive data.

***Protecting data at rest*** concerns several data types in the context of blockchain. The following bullet points elaborate on how stored data is protected by the reference architecture [38].

**Figure 9:**
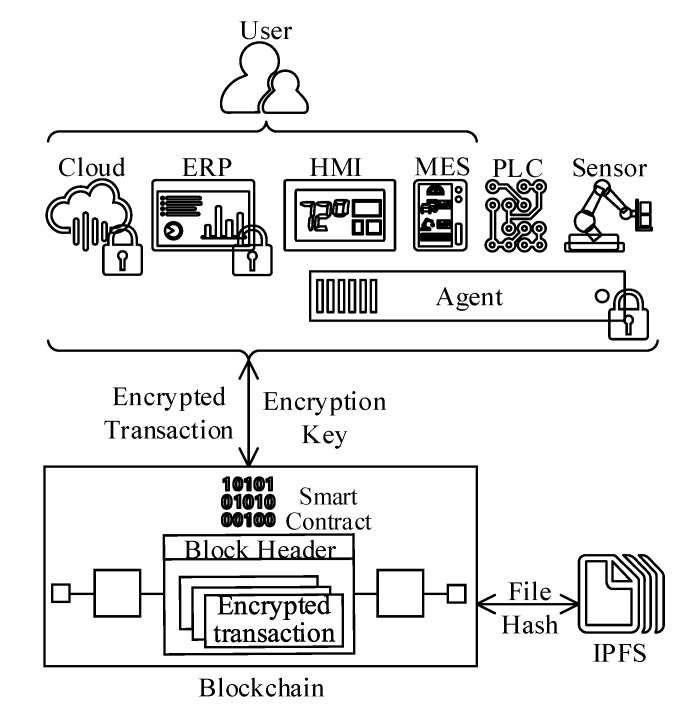
On-chain and off-chain transaction encryption.

**Figure 10:**
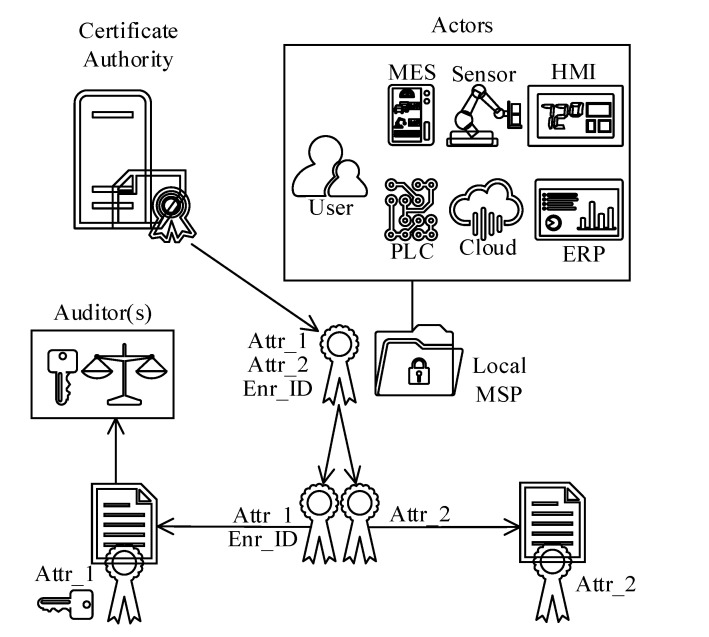
Components of IIM [38] adopted for the reference architecture to support PPABA anonymization and PPABA auditability functions.

(a) *Transactional data* constitutes the heart of the blockchain system. The reference architecture embodies four distinct security controls to protect the transactional data as follows.
  *1. Permissioned blockchain:* The first layer of data protection is ensured with the permissioned blockchain deployment topology that allows only authorized actors to access the environment. Besides, the file system encryption is enabled for all peers and nodes per default.
  *2. Channels:* The data is replicated among all peers and nodes, even with the permissioned installation. The blockchain distributed design approach leads to data confidentiality and privacy concerns. The reference architecture addresses these challenges through virtual segmentation techniques. For instance, IHF can establish multiple virtually segmented ledger instances (i.e., channels) to isolate data among ledgers. The channels are also protected with robust access control mechanisms. Thus, only authorized and explicitly privileged actors can access the data residing on the blockchain [9]. According to Chowdhury et al. (2019) [33], IHF with channel capability significantly stands out from the rest of the blockchain platforms from the privacy standpoint.
  The ISA95 functional model defines 12 core enterprise functions to organize the manufacturing process [15]. Hence, an enterprise applying the reference architecture for all core ISA95 functions should establish at least 12 channels.
  *3. On-chain transaction encryption:* There are several ISA95 business flows (e.g., “Product Cost Account 8.0” function) that process sensitive data such as trade secrets and financial transactions. For scenarios when sensitive material is processed or transported over the ledger, the reference architecture protects the data at rest with robust symmetric encryption algorithms. Figure 9 illustrates the data encryption schema for the reference architecture. The design assumption is that the ISA95 actors at level 4 and 5 can encrypt the data on the application side, whereas the rest may need an intermediary agent software for encryption.
  The data processing life cycle begins when an actor encrypts sensitive information. Next, the ciphertext is supplied to a smart-contract over an encrypted communication channel. The encrypted data is not processable; thus, the encryption key is also provided to the smart-smart contract through a particular temporary parameter for decryption. IHF characterizes this parameter with the transient term to indicate that it is purged at the end of the smart-contract execution and not stored on-chain anytime. The next step after decryption is the processing phase, during which the sensitive data is protected inside a virtual enclave environment. Finally, the execution outcome is encrypted with the provided key before being recorded to the ledger [38]. The end-to-end encryption strategy maintains the confidentiality and privacy of data at all times.
  *4. Off-chain transaction separation and encryption:* Certain regulatory requirements, such as GDPR, restrain distributing sensitive data geographically. However, modern manufacturing organizations depend on complex international engagements. Therefore, restricting the ledger deployment on a geographical basis would seriously limit the reference architecture’s applicability. Hao et al. (2020) [39] studied a similar scenario where private organizations share data with governmental agencies. In their proof of concept work, the sensitive data is distributed through a network-attached file share without applying encryption, similar to the “Use-case 3” described in previous sections.
  The reference architecture attempts to address location restrictions by preserving the sensitive data on an IPFS based network storage that is geographically situated as per regulatory requirement. However, in order to add an extra layer of protection, individual data elements are encrypted, and hash to the files are distributed through the ledger. Figure 9 depicts the architectural components of the proposed off-chain encryption solution.
  An example scenario concerning the ISA95 would be the realization of the “Production Shipping Admin 9.0” flow constituting customer data in the form of personally identifiable information (PII), which is subject to GDPR and required to remain within the boundaries of the EU.

(b) *Smart-contract and smart-contract state protection:* An ordinary blockchain system saves and replicates smart-contract logic (source code) and state data (an outcome of an execution) across the blockchain by default. In other words, the execution results, state data, and smart-contracts are visible to all peers and nodes. However, some business engagements and contractual terms are trade secrets, and unauthorized access is not acceptable. The reference architecture addresses these challenges with a two-folded approach adopted from IHF [9]. Firstly, any sensitive state data can be selectively encrypted, as shown in Figure 9. The encryption restricts access to the actors who have the decryption key. Secondly, sensitive smart-contracts can be deployed to an isolated containerized node where replication is prohibited. This deployment strategy decreases the availability of particular smart-contract in favor of increasing security.
(c) *User data protection:* Traditional blockchain technologies, such as Bitcoin, are infamous for preserving user privacy and transactional anonymity. In other words, the transactions are traceable and linkable to a user profile. The reason is that traditional blockchain technologies rely on certificate-based authentication standards, such as X.509, and the certificate-based schemas are not able to hide nonessential attributes from the certificates. Hence, user privacy is profoundly disrupted [40]. Furthermore, actors always sign transactions with the same private key, and, even if the identities are secret, all the transactions signed by the same actor are still linkable to each other. Besides, in the case of identity theft, all the transactions committed by the stolen key become fully traceable and available to the public.
  Camenisch et al. (2013) [40] proposed a novel privacy-preserving attribute-based authentication (PPABA) schema to overcome the anonymity and transaction likability shortcomings. The strength of PPABA is that the issuer can generate separate tokens for each user attribute, and a user can produce (derive) unique transactional tokens (without requiring issuer interaction) that are verifiable with the issuer’s public key. Because the user forges a new token for each transaction, it is not feasible to trace back and associate the transactions with an individual even after a private key compromise. Besides, the PPABA schema ensures higher anonymity by excluding unnecessary user attributes from the transactional data.
  Commercially available IBM Identity Mixer (IIM) and Microsoft U-Prove provide pluggable PPABA libraries that can constitute a basis to ensure user data protection for the reference architecture.
  Figure 10 illustrates the reference architecture components adopted from IIM [38] to support the PPABA schema. Each ISA95 actor in Figure 10 is assigned a membership service provider (MSP) that secures certificates and private keys. According to the example shown in Figure 10, the CA issues a certificate to an ISA95 actor with three attributes, Attr_1, Attr_2, and Enr_ID. However, because the particular transaction in question does not require all identity attributes, the actor derives an entirely new certificate token only with Attr_2 to be able to sign the transaction with minimum identity information. The most significant advantage of the PPABA schema is that the signed transaction is still verifiable with the CA’s public key, even if the signature is based on a key that is not issued by the CA.
  On the permissioned blockchain platform side, Monero anonymizes the transactional origin and destination to protect privacy. The anonymization process produces a group signature that obfuscates the signer’s identity with the CryptoNote algorithm. Furthermore, a one-time generated stealth address hides transactional sources [41].

**b.**  **Logging, traceability, and auditability:**

Audit and system logging are utterly crucial in ensuring traceability and auditability in a conventional IT ecosystem. However, the prominence of audit logging has decreased since the introduction of the BCT. This is because each data block is individually signed and chained to each other with anchoring hashes. Hence, the blockchain ensures robust and undeniable traceability and auditability without requiring any additional tooling.

On the other hand, system logging capabilities remain essential for troubleshooting, monitoring, and follow-up purposes, even with the BCT. Considering the complexity and ubiquity of the ISA95-CTS and SMMS, covering the entire ecosystem’s logging is challenging. Besides, ensuring the scalability and security of the system logging infrastructure are essential system design requirements. Given these, Schorradt et al. (2019) [15] proposed using blockchain-based log management and collection infrastructure to meet the scalability and security requirements. The solution proposed by Schorradt et al. (2019) [15] flattens the ISA95 automation pyramid and enables all ISA95 actors to transmit logs to the ledger independently. Therefore, the logging infrastructure becomes an enterprise-wide single source of truth or evidence of events across all applications. Because each log message is signed and chained to each other with hashing, log content integrity and immutability are guaranteed. Finally, the distributed architecture contributes to ensuring high availability.

In terms of the reference architecture, we propose to consolidate all system logs across the ISA95 enterprise on a dedicated blockchain instance (i.e., channel). Access to this ledger is restricted to system administrators and developers only. As a rule of thumb, the system logs should not contain any transactional or sensitive data. Thereby, all downstream systems logging towards the blockchain should scrutinize the content before the transmission. A specially crafted smart-contract can perform a log content verification for further assurance on data leak. In the case of sensitive information (such as PII), predefined masking algorithms can cleanse the logs prior to registering them to the ledger.

The auditability and traceability are among the most prominent characteristics of plain ledger-based systems, and they are supported out of the box. However, some business scenarios can demand higher confidentiality and privacy levels. This research paper proposed to address these requirements with data encryption and PPABA (under “*On-chain transaction encryption*” and “*User data protection*” sections). The challenge with both methods is that the data traceability is lost during the lifecycle. Therefore, it is not possible to audit the transactions.

The reference architecture addresses the data encryption auditability challenge for the “*On-chain transaction encryption*” case with threshold cryptography and key escrowing. The threshold cryptography consists of techniques to encrypt data with a public key, whereas the corresponding private key is split and distributed among multiple parties. The most crucial property of threshold cryptography is defining a threshold policy to decide the number of required parties to decrypt the data. In other words, as per the policy statement, “m” out of “n” persons can join their keys to decrypt the data.

The key escrowing is a technique to backup transactional encryption keys in a secure location. In order to ensure a higher level of security, the escrowing service can encrypt the transactional encryption keys with a threshold encryption technique so that the key recovery cannot be possible without multiple participants’ consent. This approach ensures four eyes and separation of duties principles.

In terms of the reference architecture, the data subject to auditing is encrypted with an escrowed key. After the encryption, the escrowing service acquires a copy of the transactional encryption key and stores it safely, where the transactional encryption key is encrypted with threshold encryption. The escrow decryption key is then split into chunks and distributed among multiple stakeholders, including auditors, etc., within the organization to ensure the separation of duties. When an audit request comes in, the required number of individuals come along and release the data decryption key to the auditor.

The reference architecture addresses the PPABA anonymized transactions auditability challenge for the “*User data protection*” case with a specially crafted auditor role defined by the IIM [38]. The enterprise CA generates private and public key pair for the individuals who are appointed as auditors. All the public keys belonging to the auditors are then made available across the blockchain network. Depending upon the audit and compliance policies, the actors are instructed to encrypt the unique ID (Enr_ID in Figure 10) of the transaction owner with the respective auditor’s public key. The encrypted ID is then set as a transaction token attribute, which is blended inside the transaction signature. With this approach, the auditor can decrypt the unique ID from the signature with the corresponding private key and associate it with an actor while the transaction is still unlikable for the rest of the blockchain participants.

The reference architecture components, enabling the PPABA auditability function, are illustrated in the bottom left corner of Figure 10.

**c.**  **Availability:**

IT systems, which are accessible from the internet, are vulnerable to availability attacks such as the denial of service (DoS) attacks. Blockchains are no exception. Saad et al. (2019) [42] identified smart-contract DoS and maliciously acting nodes as prominent blockchain attack surface against availability. Permissioned blockchains, the basis for the reference architecture, are built upon a higher trust level and not fully exposed to the internet. Therefore, the DoS attack surface is substantially smaller compared to publicly accessible alternatives.

Undeliberate hardware and software failures can also lead to availability issues. Consistency, availability, and partition fault tolerance (CAP) theorem correlates system availability, data consistency, network partitioning, and fault tolerance concerning distributed systems such as BCT [43]. According to the CAP theorem, distributed systems can only fulfill two out of the three properties stated above.

Public blockchain platforms are based upon computationally heavy consensus algorithms, such as proof of work (PoW). The CAP analysis on permissionless blockchains indicates high availability and fault tolerance by compromising consistency [43]. For instance, publicly accessible Bitcoin and Ethereum blockchain platforms harness PoW based consensus algorithms. They are characterized by their ability to withstand availability attacks with their highly distributed P2P architectures [33].

The reference architecture is designed to make use of the RAFT consensus algorithm. The rationale behind the design decision is that RAFT is a deterministic, fast, and efficient consensus algorithm that maintains low latency and high system throughput. Thereby, RAFT is considered the best fit for permissioned blockchain architectures. RAFT’s CAP analysis indicates high data consistency and fault tolerance (crash tolerant), whereas moderate system availability [43].

IBM proposes to increase the availability of permissioned blockchain (IHF with RAFT consensus algorithm) implementations with the following high availability deployment models [44]:

(a) Single region, single availability zone, and single worker node
(b) Single region, single availability zone, and multiple worker nodes
(c) Single region, multi-availability zones, and multiple worker nodes
(d) Multi-region, single availability zone, and multiple worker nodes

A region represents a geographical area containing a set of data centers grouped as availability zones within the same region. In other words, each region consists of multiple physically isolated availability zones. The worker nodes are provisioned on physical hardware in the form of virtual machines. IBM recommends distributing the worker nodes across availability zones to avoid worker node failures.

IBM suggests deployment options “c” and “d” to meet the production-grade availability requirements. Because the likelihood of losing an entire region is remarkably low, and inter-region communication is prone to higher latency, the reference architecture is designed upon high availability deployment option “c.”

Elimination of a single point of failure is the fundamental design criteria for high availability. In other words, all critical system components should be redundant to sustain availability in case of a failure. Referring to the logical view (Figure 1), ledger, enterprise, and integration tiers contain the most crucial blockchain reference architecture elements. The following paragraphs elaborate on the conditions to ensure high availability on these tiers.

Starting with the ledger tier, IBM [44] suggests allocating at least five dedicated nodes (aka ordering service) to operate the consensus protocol. This is because the ordering service constitutes the heart of the blockchain infrastructure. Moreover, all channel instances conceptually share the ordering service. Therefore, at least five nodes must be employed in multiple availability zones to guarantee crash fault tolerance.

The rest of the blockchain nodes (peers) should ensure the duality principle (two instances) per client application type for each channel. Referring to the logical view (Figure 1), the cloud tier and the device tier have one each, the edge tier has three, and the enterprise tier has two client application types (seven in total). In other words, the reference architecture requires at least fourteen peers to guarantee high availability.

**Figure 11:**
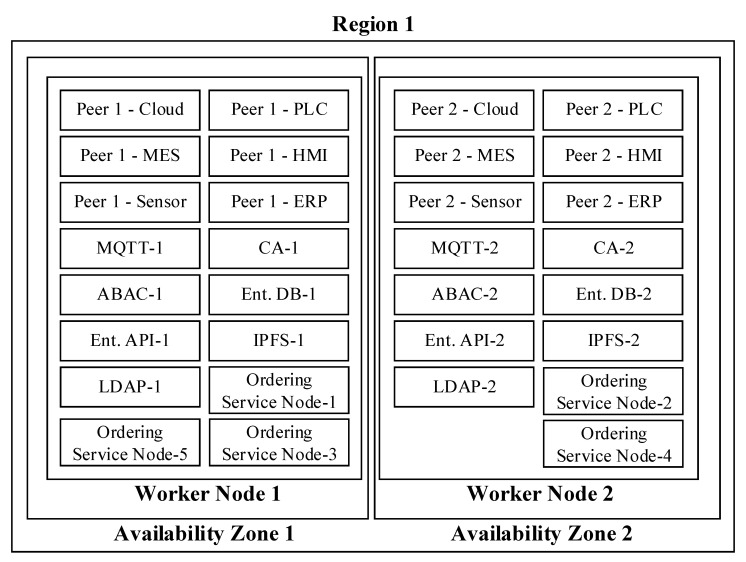
Reference architecture high availability deployment components.

The duality principle also applies to the system solutions residing inside the integration and enterprise tiers. The reference architecture identified MQTT, ABAC, enterprise DB, enterprise API, CA, LDAP, and IPFS as the supporting systems to maintain blockchain stability and functionality. Figure 11 illustrates these systems and the other blockchain critical system components from the high availability perspective. A single region with two availability zones constitutes two separate worker node installations where the vital blockchain systems are blended.

**d.**  **Identity and Access Management (IAM):**

IAM has been playing a central role in ensuring the security of enterprise infrastructure systems. In fact, identity has been promoted as a new security perimeter to protect enterprise resources in recent years.

Traditional enterprise IAM systems identify users with a unique identifier. These identifiers, user privileges, and attributes are centrally stored in enterprise identity repositories such as LDAP. To simplify user and access management, the enterprise applications (such as ERP) integrate with LDAP and fetch all user attributes from the central repository.

The functional requirements section identified and explained four generic blockchain communication use-cases, and one of which is about ledger-to-IPFS communications. According to this scenario, large files are distributed through IPFS over the P2P network. IPFS does not dictate any access control by default. The ISA95 enterprise is a heterogeneous environment with many actors. Thereby, the IPFS based enterprise file share should be protected from unauthorized users. OAuth, SAML, and Kerberos-based single sign-on (SSO) and authentication schemas are among the most popular technologies applied to IPFS scenarios. In fact, Hao et al. (2020) [39] studied a similar use-case with IPFS and blockchain and proposed to authenticate the users with OAuth authentication schema. The reference architecture employs an enterprise-wide LDAP that can produce Kerberos authentication tickets, which is the basis for controlling access on IPFS. Furthermore, user privileges are managed and leveraged with role-based access control (ABAC) through LDAP.

The permissionless blockchain platforms (such as Bitcoin, Ethereum, EOS, and Cardano) rely on pseudonymous identification methods via public keys. Hence, a foundational IAM capability is not necessary. However, permissioned blockchain platforms need to register and identify each user explicitly [30]. Thereby, flexible and robust IAM capabilities become a crucial success factor in enabling blockchain technology adaptation.

The permissioned blockchains, such as IHF and Sawtooth, deviates from the traditional IAM model for the sake of extensibility. The identities in IHF are managed via dedicated MSPs, which are attached to each blockchain peer, node, and channel [45]. In a consortium blockchain spanning across multiple organizations, each organization operates a dedicated MSP to administrate identities. Having dedicated MSP per organization establishes an abstraction layer between the blockchain and the organizational IAM operations, which unify the IAM operations across the ledger.

The reference architecture model is fundamentally designed over the standard IHF IAM capabilities. Beyond the conventional IHF features, the reference architecture is enriched with role-based access control (RBAC) and attribute access control (ABAC) capabilities.

Each actor illustrated in Figure 1 talks to the blockchain through a client application, which can be in various forms. For instance, the cyber-physical devices located in level 0, 1, 2, and 3 can exchange data with the ledger through an interceptor agent. In comparison, layer 4 and 5 applications, such as ERP, can directly transmit data to the blockchain. Thereby, the IAM model of the reference architecture should be flexible and versatile enough to identify and manage a broad range of ISA95 actors with different capabilities.

The reference architecture is designed on certificate-based identification and authentication services. In other words, each ISA95 actor is assigned a unique private key and a public key in the X.509 certificate format, which are issued by the enterprise certificate authority (CA). The private key is the basis for identification and employed to sign and secure individual transactional data. Given these critical functionalities, the private key’s integrity and confidentiality are crucial aspects of the reference architecture. Thereby, these keys should be protected in tamper-proof MSP environments, such as software-based Keystore, hardware-based security modules (HSM), and smart cards.

Besides the ISA95 actors, the blockchain infrastructure elements, such as transaction validation and ordering service nodes, are also assigned dedicated MSPs to protect the service identities. This delicate IAM architecture with extensive service identification capabilities ensures high accountability and traceability at every stage of the ledger data life cycle.

The MSP service not only plays a significant role in managing identities but also provides access control functionality. The channel MSPs embody a list of authorized actions (i.e., read and write) assigned to specific actors. Moreover, a list of privileged users, administrators, are also available to the channel MSPs.

Transaction validation nodes contribute to the access control functionality with the following validation and confirmation operations.

- The transaction validation nodes make sure that the signer’s public key is not in the certificate revocation list (CRL), and the signature is computationally correct.
- The transaction validation nodes confirm that the signer is authorized to perform the demanded operation. Furthermore, the MSP access policy can leverage the attributes defined by the signer’s public certificate. However, some complex MSP policies may require additional attributes outside the standard X.509 certificate format. In such scenarios, the certificate content can be enriched with the information ingested from the enterprise LDAP. The externally provided LDAP attributes can contain a broad range of information, including group memberships (access privilege) of actors. Hence, the access control capability of the reference architecture is extended to support the RBAC model.

**Figure 12:**
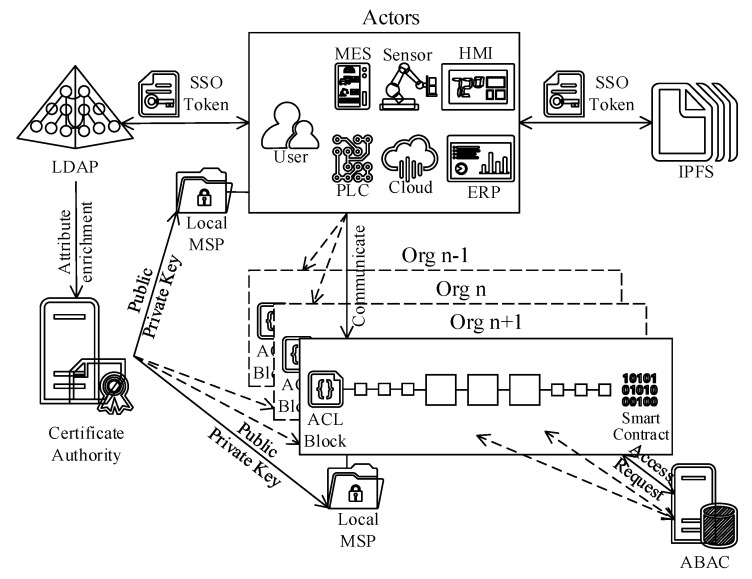
Reference architecture IAM components.

Coarse-grained access control capabilities presented via MSP and RBAC models are an excellent fit to realize most ISA95 IAM scenarios. However, some ISA95 enterprise functions might demand context-aware and finer-grained access control capabilities. The reference architecture proposes to realize these requirements by adopting the attribute-based access control (ABAC) model for the ledger ecosystem. Our previous research paper studied the ABAC model’s feasibility and applicability for the industrial control systems (ICS) and implemented a POC application [46]. The ABAC model features context-aware, dynamic, flexible, and centrally auditable fine-grained access control capabilities. A traditional ABAC infrastructure is composed of several components. However, the policy decision point (PDP) and policy enforcement point (PEP) are crucial components of the blockchain reference architecture. The fine-grained access control policies are implemented with the XACML language and executed on PDP. PEP sends and receives access requests and responses from/to PDP. Smart-contracts interface the external environment through Oracles. Thus, a smart-contract developed to perform access control functionalities can interact with the external ABAC infrastructure through PEP, which merely acts as a data Oracle for the blockchain environment. An example access request could be “Sensor XYZ would like to write temperature information to the ledger on channel ABC on Sunday at 03:28.” Depending upon the access policy, PDP can respond with either a “permit” or “deny” message. Figure 12 demonstrates various components of the IAM capability for the reference architecture.

### 3.4. Soft Dimensions of Reference Architecture

The following categories briefly elaborate on the soft dimensions of the reference architecture:

- *Viability:* Ivanov and Dolgui (2020) [3] researched the effects of the COVID-19 outbreak on the global supply chain networks. The COVID-19 pandemic has sadly shown the fragility of the supply chains under extreme conditions. This is because the modern supply chains are either designed in linear or network topology. Ivanov and Dolgui criticized that most supply chains are designed to maintain high production output and performance. Thereby, the supply chains usually disregard the survivability aspect to ensure society’s mobility and communication under extraordinary situations. Ivanov and Dolgui proposed the intertwined supply network concept that constitutes highly interconnected entities, guaranteeing the provision of goods to the communities in any condition.
  Lack of trust among suppliers and infrastructure fragility are two main challenges affecting supply chain viability. Kumar et al. (2020) [47] highlighted how blockchain-based supply chain platforms could establish trust among untrusted suppliers by ensuring high transparency. Likewise, we elaborated in the availability section on how the reference architecture is designed to withstand significant disruptions with robust infrastructure deployment options. Thereby, the reference architecture can significantly improve supply chain viability.

- *Sustainability:* The importance of sustainability has become significant in recent years, especially since UN member states’ SDG adaptation. Leng et al. (2020) [4] studied SDGs and correlated “Goal 12: Responsible consumption and production” and “Goal 9: Industry, innovation, and infrastructure” with sustainable manufacturing practices. They also identified that distributed manufacturing, including crowd and clustered manufacturing, are the primary driver for these goals and surveyed different BCTs available in the literature from the manufacturing sustainability perspective. The survey conducted by Leng et al. highlighted that the BCT could enhance efficiency, reduce the system complexity with high interoperability, increase the manufacturing system security, boost operational transparency with traceability and ensure end-product authenticity.
  Jabbour et al. (2020) [48] surveyed the sustainability aspects of various supply chain networks. They concluded that BCT and big data analysis methods could improve the agriculture industry’s efficiency by shortening the supply-chain networks. Likewise, BCT could enhance the accuracy of procurement forecasts in the agriculture industry.
  Wamba et al. (2020) [49] empirically explored dynamics between blockchain determinants and supply chain performance. Their research justifies the positive relationship between blockchain adaptation and supply chain performance through increased transparency.
  In a nutshell, the reference architecture’s design specifications are compliant with the BCT empowered sustainability outcomes presented by multiple researchers. Thereby, the reference architecture constitutes a crucial blueprint to enable the manufacturing industry to meet UN SDGs.

- *Social and economic inclusion:* Several rural societies are disconnected from developed cities. Due to insufficient economic opportunities, the gap between developed and underdeveloped regions is getting wider. Thereby, social and economic inequality has become prevalent in developing countries. Schuetz and Venkatesh (2020) [5] studied India and financial inclusion from this perspective. Their research highlights that rural India can gain momentum on economic and social development by expanding global supply chain networks to the remote villages. Schuetz and Venkatesh also proposed to adopt blockchain-based technologies to extend the global supply chain coverage towards the rural areas. Thereby, blockchain-enabled ISA95 enterprise functions focusing on supply chain use cases can support resolving the social and economic inclusion issues in developing countries.

## 4. Conclusions

The industry 4.0 revolution has deeply impacted traditional manufacturing and production industries relying upon legacy standards such as ISA95. Although new-generation IT technologies significantly penetrated the industry with smart manufacturing concepts, outdated systems still prevail. Hence, ISA95-CTS and SMMS are inevitably required to collaborate and integrate with each other to constitute the highly complex and heterogeneous modern manufacturing ecosystems.

Our former research article focused on the contemporary manufacturing industry and identified systems scalability, interoperability, information security, and data quality domains as the significant challenges profoundly impairing the productivity of ISA95-CTS and SMMS [1]. Furthermore, we analyzed BCT’s proposition and proposed to empower the ISA95-CTS and SMMS with BCT to address the identified challenges.

We also meticulously assessed BCT’s suitability for the manufacturing processes defined by the ISA95 standard as part of another research work [2]. We identified that 49 out of the 85 sub-functions and 7 out of the 12 core functions defined by the ISA95 standard are suitable for applying BCT.

Besides technical prospects, we also justified how the reference architecture can foster supply chain viability, sustainable development in manufacturing, and social and economic inclusion in rural areas.

Despite the BCT proposition to empower the manufacturing industry, our literature survey could not identify a reference architecture focusing on ISA95-CTS and SMMS. The mentioned scientific gap constitutes the foundation of this research effort.

The reference architecture in this research is developed by following the IEEE 1471 software architecture standard principles. Firstly, the outcomes of previously committed studies formed the basis for defining the functional and non-functional system requirements. Secondly, the reference architecture logical view and corresponding components are specified. Lastly, each system requirement is methodically analyzed, and thorough literature research is conducted to identify building blocks of the reference architecture to realize the requirements, as shown in Table 1.

Our study concludes that the proposed reference architecture is equipped with all necessary technologies and system components to meet or even exceed the ISA95-CTS and SMMS requirements.

## 5. Future Work

Modern system architecture designs are composed of several views to address all aspects of an enterprise application. The reference architecture only covers the logical view. Thus, a prospect research paper(s) may focus on other enterprise architecture views. Therefore, the blockchain reference architecture for the ISA95-CTS and SMMS will evolve towards an enterprise blockchain architecture model for the ISA95-CTS and SMMS. In this context, the implementation view is specifically essential to produce and collect quantitative scientific results, which will be the basis to confirm the reference architecture’s applicability.

Moreover, this research paper proposes to leverage various cutting-edge technologies that have not been researched in the BCT context. For instance, extending the access control capabilities of BCT to support ABAC through an off-chain PDP is an entirely new proposition that has not been studied before.

Lastly, this research paper briefly listed three main categories of soft dimensions of adopting the reference architecture, which can be explored further as individual research topics.

## Figures and Tables

**Table 1:** Design requirements versus building blocks of the reference architecture.

	Requirements		Requirement Specifications	Realization of Requirements
Functional requirements	Regulatory compliance		Ensure regulatory compliance	1. Smart-contract implementation of compliance requirements 2. Dedicated state database to safeguard compliance parameters 3. Regulators and auditors are provided with a read-only interface to perform compliance verification activities
	Data integrity and signing		Support a broad range of data integrity and digital signature schemas	1. Tamper-proof data blocks linked to each other with a hash-based pointer 2. Pluggable cryptographic library extensions to support various blockchain signature algorithms such as aggregate, multi-signatures, and privacy-preserving NI-ZKP 3. Off-chain data hash and signatures are stored on the ledger
	Automation		Promote and allow manufacturing automation capabilities	1. Deterministic process automation with tamper-proof smart-contract execution 2. Smart-contracts emit event messages to contribute autonomous decision mechanisms
Non-functional requirements	Scalability		Ensure horizontal and vertical scalability	1. A fully virtualized container environment ensuring horizontal and vertical scalability for processing capacity and storage area 2. Big files are stored on IPFS to reduce ledger footprint
	Performance		Keep latency below 300 ms and ensure 6000 to 8000 tps	1. Permissioned ledgers with trivial RAFT consensus algorithm exceed 10,000 tps 2. At least four CPU instances per ordering service 3. At least four individual ordering nodes 4. Block size of 80 transactions to ensure latency
	Interoperability		Ensure interoperability and seamless integration across ISA95 automation layers	1. MQTT based integration layer ensures technical and syntactic interoperability 2. Systems compliant with ISA95 standard are already semantically interoperable3. Smart-contracts ensure governance interoperability
	Data quality		Define a set of quality violation policies as unified system behaviors	1. Accept and log on violation policy when a quality parameter is not in the range 2. Alarm on violation policy when a quality parameter is outside the range 3. Raise an event on violation policy to correct the situation 4. Defer decision on violation policy when a single violation is not sufficient to make a concrete decision
	Information Security	Data protection	Protect data at rest, in transit, and during processing from unauthorized access	1. Protecting data in transit with TLS 2. Protecting data while in use with Containerized TEE technology 3. Protecting data at rest through permissioned blockchain and virtual segmentation techniques. 4. Robust symmetric encryption algorithms to protect on-chain data, smart-contract state data, and IPFS at rest 5. Isolated containerized nodes to protect smart-contract source code during deployment 6. PPABA schema to overcome anonymity and transaction unlikability challenges
		Logging, traceability, and auditability	Ensure traceability and auditability along with robust, secure, and scalable logging services	1. Consolidated system logs across on a dedicated blockchain instance 2. Restricted system log access and sensitive information masking 3. Threshold cryptography and key escrowing to allow auditability for the on-chain encrypted data 4. Issuer ID encryption with auditor public key to allow PPABA anonymized transactions auditability
		Availability	Ensure high availability and system robustness	1. RAFT crash tolerant consensus algorithm 2. High availability ensured with a single region, multi-availability zones, and multiple worker node deployments 3. At least five dedicated nodes to operate RAFT 4. All peers ensure duality principle per client application type for each channel
		Identity and access management	Provide capabilities to identify, authenticate, and authorize actors and embody technical mechanisms to ensure fine-grained authorizations	1. Access to IPFS is protected with SSO based protocols such as OAuth and SAML 2. IAM operations are managed on the blockchain ecosystem through MSP 3. Certificate-based identification and authentication services 4. MSP content is protected with HSM, Keystore, and smart cards 5. RBAC and ABAC are supported for the scenarios where fine-grained access control capabilities are demanded
